# Machine learning models for diagnosis and risk prediction in eating disorders, depression, and alcohol use disorder

**DOI:** 10.21203/rs.3.rs-3777784/v1

**Published:** 2024-02-01

**Authors:** Sylvane Desrivières, Zuo Zhang, Lauren Robinson, Robert Whelan, Lee Jollans, Zijian Wang, Frauke Nees, Congying Chu, Marina Bobou, Dongping Du, Ilinca Cristea, Tobias Banaschewski, Gareth Barker, Arun Bokde, Antoine Grigis, Hugh Garavan, Andreas Heinz, Rudiger Bruhl, Jean-Luc Martinot, Marie-Laure Paillère Martinot, Eric Artiges, Dimitri Papadopoulos Orfanos, Luise Poustka, Sarah Hohmann, Sabina Millenet, Juliane Fröhner, Michael Smolka, Nilakshi Vaidya, Henrik Walter, Jeanne Winterer, M. Broulidakis, Betteke van Noort, Argyris Stringaris, Jani Penttilä, Yvonne Grimmer, Corinna Insensee, Andreas Becker, Yuning Zhang, Sinead King, Julia Sinclair, Gunter Schumann, Ulrike Schmidt

## Abstract

This study uses machine learning models to uncover diagnostic and risk prediction markers for eating disorders (EDs), major depressive disorder (MDD), and alcohol use disorder (AUD). Utilizing case-control samples (ages 18-25 years) and a longitudinal population-based sample (n=1,851), the models, incorporating diverse data domains, achieved high accuracy in classifying EDs, MDD, and AUD from healthy controls. The area under the receiver operating characteristic curves (AUC-ROC [95% CI]) reached 0.92 [0.86-0.97] for AN and 0.91 [0.85-0.96] for BN, without relying on body mass index as a predictor. The classification accuracies for MDD (0.91 [0.88-0.94]) and AUD (0.80 [0.74-0.85]) were also high. Each data domain emerged as accurate classifiers individually, with personality distinguishing AN, BN, and their controls with AUC-ROCs ranging from 0.77 to 0.89. The models demonstrated high transdiagnostic potential, as those trained for EDs were also accurate in classifying AUD and MDD from healthy controls, and vice versa (AUC-ROCs, 0.75-0.93). Shared predictors, such as neuroticism, hopelessness, and symptoms of attention-deficit/hyperactivity disorder, were identified as reliable classifiers. For risk prediction in the longitudinal population sample, the models exhibited moderate performance (AUC-ROCs, 0.64-0.71), highlighting the potential of combining multi-domain data for precise diagnostic and risk prediction applications in psychiatry.

**Additional Declarations: Yes** there is potential Competing Interest. Dr Banaschewski served in an advisory or consultancy role for Lundbeck, Medice, Neurim Pharmaceuticals, Oberberg GmbH, Shire. He received conference support or speaker's fee by Lilly, Medice, Novartis and Shire. He has been involved in clinical trials conducted by Shire & Viforpharma. He received royalties from Hogrefe, Kohlhammer, CIP Medien, Oxford University Press. Dr Barker has received honoraria from General Electric Healthcare for teaching on scanner programming courses. Dr Poustka served in an advisory or consultancy role for Roche and Viforpharm and received speaker's fee by Shire. She received royalties from Hogrefe, Kohlhammer and Schattauer. M. John Broulidakis receives a salary from medical device manufacturer Emteq Labs for which he works as a research scientist. Emteq Labs had no role, financial or otherwise, in the STRATIFY or IMAGEN projects or this paper in particular. Views expressed in this paper do not necessarily reflect those of Emteq Labs. The present work is unrelated to the above grants and relationships. The other authors report no biomedical financial interests or potential conflicts of interest.

## Main text

Eating disorders (EDs), including Anorexia Nervosa (AN), Bulimia Nervosa (BN), Binge Eating Disorder (BED) and related sub-clinical syndromes, are a major healthcare challenge, with significant public health and economic impacts. These complex and disabling disorders affect 6–18% of young women and up to 2% of young men by early adulthood [1]. With a typical age of onset of between 15 and 25 years, EDs seriously impact young people's life chances, their families, and the wider society [2]. Mortality rates in people with EDs are twice as high as in the general population, and about six time higher for people with AN [3]. Psychiatric comorbidities such as anxiety, mood, and substance use disorders are common and negatively impact ED outcomes [4]. This complexity makes EDs hard to detect and treat, and relapse occurs frequently [5]. Early detection and more accurate patient classification are key priorities in the development of effective interventions.

A multifactorial neurodevelopmental model has been proposed to explain the complexity of EDs [6]. Widely accepted risk factors include sex, body mass index (BMI), weight/shape concerns, low self-esteem, a history of depression, anxiety, attention-deficit/hyperactivity disorder (ADHD) symptoms, and disordered eating behaviors [7]. Personality traits, notably neuroticism, have also been implicated in EDs [8]. At the environmental level, traumatic experiences of neglect and abuse in childhood are linked to higher risks of ED pathology [9,10]. However, while there is evidence for multiple biopsychosocial risk factors, most studies typically focus on only a single or a small number of risk factors. It is still unknown which combinations of factors will most accurately reflect ED susceptibility/risk or improve diagnostic classification, which is a focus of the current study.

Over half of individuals with EDs have a co-occurring psychiatric disorder, with anxiety and mood disorders being the most prevalent, both affecting over 50% of individuals with EDs [11]. Alcohol use disorder (AUD) affects about one in five individuals with EDs [12]. Recent studies have shed light on the common genetic [13,14] and neural [15] underpinnings of these conditions, indicating shared underlying mechanisms. The current study aims to identify psychosocial correlates and early risk factors that are shared and specific across EDs, major depressive disorder (MDD), and AUD.

Machine learning methods and the emergence of large data cohorts have provided opportunities to build multivariate risk profiles for psychiatric disorders. In ED research, these have been used in cross-sectional diagnostic classification models derived from distinct datasets, such as questionnaires [16,17], social media [18], or neuroimaging data [19,20]. Longitudinal models have also been built to predict illness course [21] and treatment outcomes [22]. Yet, to our knowledge, no ED study to date has combined a wide range of data domains to build models for diagnostic classification or risk prediction.

We addressed this research gap by deriving machine-learning-based models from broad domains of psychosocial data, collected from two samples that underwent similar assessments: 1) a clinical sample comprising people with AN, BN, MDD, and AUD, and 2) a longitudinal population-based cohort of adolescents followed from ages 14 to 19 years. Analyses in the clinical sample were conducted with the aim to identify multidomain markers for diagnostic prediction of EDs, MDD, and AUD, and describe their most important classifiers. Analyses in the longitudinal population sample aimed at identifying reliable markers for susceptibility/risk of developing symptoms of EDs, MDD, and AUD.

### Results

## Characteristics of the samples

In the clinical sample, mean ages ranged from 22.02 to 22.74 years across participant groups. The AN (N = 62) and BN (N = 50) groups and their corresponding controls (N = 57) were all female. The MDD (N = 176) and AUD (N = 159) groups and their controls (N = 99) involved 75%, 58%, and 59% female participants, respectively (Supplementary Table 1). All the clinical samples were Caucasian, except for the control group for AN and BN (81.1% were Caucasian). In the longitudinal population sample, 1,851 participants (47.4% being female, 88.9% being Caucasian) completed the initial assessment at age 14 years and at least one of the two follow-up assessments at ages 16/19 years. From these, we identified developers of ED symptoms (N = 221, 59% female) and controls (N = 511, 30% female) who remained asymptomatic across the three ages. We also identified 271 developers of depression (62% female) and their 798 controls (46% female), and 522 developers of harmful drinking (39% female) and their 806 controls (55% female). Percentages of missing data are provided in Supplementary Tables 2–4.

## Modeling current EDs

Analyses involving all data domains (47 variables) yielded near-perfect classification performance, as measured by area under the receiver operating characteristic curve (AUC-ROC [95% CI]): AN vs HC: 0.97 [0.94-1.00], BN vs. HC: 0.90 [0.83–0.96], AN vs. BN: 0.89 [0.82–0.95]. Expectedly, the high accuracy of classifying AN against the other two groups was dominated by the inclusion of BMI. Re-running all analyses excluding BMI still yielded a very high AUC-ROC (0.92 [0.86–0.97]) for AN vs. HC classification, indicating that variables other than BMI can accurately classify AN. For AN vs. BN, the AUC-ROC dropped to 0.75 [0.65–0.83] without BMI but remained significant ($p < 2.0E-04$), while for BN vs. HC, AUC-ROC was 0.91 [0.85–0.96], indicating that BMI did not contribute at all to this classification (Fig. 1, Supplementary Fig. 1). Additional model performance metrices, including area under the precision and recall curve (AUC-PR), sensitivity, specificity, precision, and recall are provided in Supplementary Tables 5–6.

Re-running analyses with each data domain separately indicated that all domains were accurate classifiers on their own (Fig. 1). Personality distinguished all three groups with good accuracies (AUC-ROCs [95% CIs], 0.77–0.89 [0.68–0.95]). Substance use could also distinguish the three groups significantly above chance, albeit with lower accuracies (0.62–0.76 [0.51–0.85]). Psychopathology, environment, and cognition distinguished AN and BN from HC (0.67–0.86 [0.56–0.93]), but not AN from BN (0.47–0.49 [0.36–0.60], Supplementary Table 5).

——Figure 1——

We extracted the top 10 reliable features from models including all the data domains except BMI. The features distinguishing both AN and BN from HC included higher neuroticism, hopelessness, symptoms of ADHD and obsessive-compulsive disorder (OCD), and poorer spatial working memory strategies (Fig. 2, Supplementary Table 7). The other reliable features distinguishing AN from HC were lower extravagance, executive function and decision making, including more working memory errors, delay aversion, risk taking, and overall proportion of bets. The other reliable contributors to BN vs. HC classification included symptoms of generalized anxiety disorders (GAD), specific phobia, drug use, and physical neglect. The AN vs. BN analysis identified six reliable features: patients with BN presented higher impulsivity, openness, extravagance, disorderliness, exploratory excitability, and drug use.

—— Fig. 2——

## Modeling current MDD and AUD

Both MDD (AUC-ROC [95% CI], 0.91 [0.88–0.94]) and AUD (0.80 [0.74–0.85]) could be distinguished from HC with high accuracies (Supplementary Fig. 2, Supplementary Table 8). To avoid circular analysis, depressive and emotional symptoms were excluded from the MDD vs. HC classification, and the harmful drinking scale was excluded from AUD vs. HC classification. In addition, variables within the cognitive domain and those measuring experiences of neglect and abuse were excluded due to excessive and imbalanced missing data across groups (Supplementary Table 3), leaving 35 and 36 predictors for MDD vs. HC and AUD vs. HC analyses, respectively. Eight and ten features reliably contributed to the accurate classification of MDD and AUD, respectively (Fig. 3, Supplementary Table 9). Five of these reliably classified both disorders from HC, including higher neuroticism, hopelessness, symptoms of ADHD and GAD, and drug use. Interestingly, neuroticism, hopelessness, and ADHD symptoms were also among the most contributing features distinguishing both AN and BN from HC. Besides these, reliable features of MDD included OCD symptoms, peer relationship problems, and harmful drinking, while those of AUD included extravagance, disorderliness, impulsivity, depression, and emotional symptoms (Supplementary Table 9).

——Figure 3——

## Transdiagnostic model performance

We investigated whether the classification models could differentiate between patients and healthy controls (HCs) in a transdiagnostic manner. The models obtained from AN vs. HC and BN vs. HC analyses accurately classified MDD and AUD from HC (AUC-ROCs, 0.75–0.93; *ps* < 2.0E-04 from permutation tests, Supplementary Table 10). The converse was also true: models developed for MDD vs. HC and AUC vs. HC classifications could accurately distinguish AN and BN from HC (AUC-ROCs, 0.83–0.92; *ps* < 2.0E-04 from permutation tests).

## Predicting the development of mental health problems

We next tested if the reliable features identified above, when assessed at age 14 in a longitudinal sample, predicted future onset of ED symptoms, depression, and harmful drinking. Emotional neglect, physical neglect, and emotional abuse were not available at age 14, and therefore excluded from analyses. Depressive and emotional symptoms at age 14 were excluded from predicting future depression, and the harmful drinking scale at age 14 was excluded from predicting future harmful drinking. In addition, we excluded three cognitive variables due to excessive missing data: delay aversion, overall proportion of bets, and risk taking, all from the Cambridge Gambling Task. This left 18, 17, and 16 predictors in the models for ED symptoms, depression, and harmful drinking, respectively. Elastic Net models were constructed on the population samples by using the same procedure as in the clinical samples.

The performance was significantly above chance for predicting future ED symptoms (AUC-ROC [95% CI], 0.64 [0.60–0.68]), depressive symptoms (0.62 [0.58–0.66]), and harmful drinking (0.64 [0.61–0.67], Fig. 4A, Supplementary Table 11). Reruning analyses after adding three known ED risk factors, namely, sex, BMI, and pubertal development, the model's performance for predicting future ED symptoms increased to 0.71 [0.67-0,75], while there was only a minor performance increase in predicting depressive symptoms (0.64 [0.60–0.68]) and harmful drinking (0.67 [0.64–0.70], Supplementary Fig. 3).

The most reliable predictors for future ED symptoms were being female, having a higher BMI, more advanced pubertal status, symptoms of depression, specific phobia, OCD, emotional symptoms, harmful drinking, and impulsivity. Particularly, impulsivity was a common reliable predictor of future symptom onset for all three disorder groups. Emotional symptoms were not included in the analysis predicting depression, but it was a common reliable predictor of ED symptoms and harmful drinking. Being female, more advanced pubertal status, and specific phobia symptoms were shared predictors of ED symptoms and depression. ADHD symptoms were shared predictors of depression and harmful drinking. The other reliable predictors of depression were higher peer relationship problems, neuroticism, and GAD symptoms. On the country, lower peer relationship problems were among the top predictors of future harmful drinking, and the other top 10 predictors included drug use, disorderliness, exploratory excitability, hopelessness, and a higher BMI (Fig. 4B, Supplementary Table 12).

——Figure 4——

### Discussion

Our multi-domain analyses combining a wide range of data from clinical and population samples have identified psychosocial profiles predictive of current and future EDs, MDD, and AUD. The classification models built for one disorder were also highly discriminative for the others, indicative of their transdiagnostic potential. Features that distinguished cases from controls also predicted future onset of ED symptoms, depression, and harmful drinking in a longitudinal adolescent sample. These results demonstrate the value of a multi-domain analysis in predicting both current and future mental illnesses. They also point towards factors that could enhance the effectiveness of early intervention and prevention strategies.

## Classification of current AN and BN

While BMI contributed most to the AN classification, performance of our models was not diminished by excluding BMI. In this respect, our "AN profile" may be a key tool to help eliminate the reliance of healthcare professionals on BMI for AN diagnosis, which has been decried for delaying diagnosis and getting in the way of early intervention [23]. In fact, DSM-5 now includes a diagnosis of atypical AN where BMI is within or above normal range. Our findings that neuroticism and hopelessness are significantly elevated in EDs corroborated previous findings [8]. Hopelessness and depression are significant predictors of suicidal ideation, attempts, and death [24]. Higher hopelessness may explain the high risk of suicide among patients with EDs [25]. Depression, hopelessness, and suicidal thoughts are common in severe and enduring AN, but in contrast to MDD, antidepressants are not particularly effective in AN. Thus, exploration of novel approaches to treatment aimed at improving mood and building hope, e.g., non-invasive neuromodulation, is urgently needed [26].

Features that distinguished AN from BN corroborate the well-established knowledge that substance use is particularly common in BN [27], and that impulsivity [8] and novelty seeking [28,29], including disorderliness, extravagance, and exploratory excitability, are shared features of BN and substance use disorders. These features may be helpful for improving stratification of AN and BN, and inform the temperament-based treatment for eating disorders [30].

## Classification of current MDD and AUD

The models trained to distinguish EDs from healthy controls were also accurate at classifying AUD and MDD, and vice versa, indicating a high degree of transdiagnostic potential. High neuroticism, hopelessness, and ADHD symptoms characterized all four disorders. The associations between neuroticism, hopelessness, ADHD, and psychiatric disorders have been implicated by previous research investigating each disorder separately. For the first time, we provide evidence for these shared associations in the same study. Genetic associations have been implicated between neuroticism, ADHD, and MDD [31], and between ADHD and EDs [32]. Similar neurobiological alterations in the executive/inhibition and reward systems have been found for ADHD, AUD, and EDs [33–35], suggesting shared mechanisms underlying these conditions. On the other hand, the different patterns observed in the psychosocial profiles across disorders highlight the uniqueness and complexity of their shared mechanisms [13]. Further research is needed to elucidate more detailed mechanisms underlying these mental illnesses.

### Predictors of future mental health symptoms

The ability of reliable disease classifiers to predict later onset of mental health symptoms is indicative of their potential in targeted prevention. Adding well-known ED related predictors specifically improved prediction accuracies for ED symptoms, which highlights the importance of feature selection in predictive modeling. Consistent with previous research, being female, depressive symptoms, a higher BMI, and pubertal development were among the most potent risk factors for developing ED symptoms [36]. Interestingly, pubertal development predicted both future ED and depressive symptoms, which might reflect the impact of being overweight/obese on puberty onset in girls, via the trigger of neuroendocrine processes [37]. A psychosocial process may also play a role: early onset of puberty for young girls confers risk for bullying and harassment [38], which in turn contributes to development of a negative body image, disordered eating behaviors, and depression [39]. This calls for early, pre-pubertal interventions in high-risk groups, such as girls with higher BMI, to prevent disease onset [40].

Higher impulsivity not only correlated with BN and AUD diagnoses, but also predated the development of ED symptoms and harmful drinking. This result suggests that impulsivity may present a common predisposition for these two symptoms to develop. Furthermore, we also identified a temporal relationship indicating that harmful drinking at age 14 years increased the risk of future ED symptoms. To date, there have been limited longitudinal studies examining the relationship between EDs and AUD, with emerging evidence indicating that ED symptoms are associated with subsequent alcohol problems [41]. Our findings, combined with this evidence, suggest a potentially bidirectional relationship between symptoms of EDs and AUD. In summary, there is evidence supporting both a shared etiological model and a causal relationship model (i.e., one disorder causes another) between EDs and AUD [42]. These findings point towards the need for integrated treatment and prevention strategies that address EDs and AUD simultaneously.

There has been consistent evidence showing that impulsivity is higher individuals with MDD and is positively associated with depressive symptoms [43], but evidence for longitudinal relationship has been limited [44,45]. Our results indicate that higher impulsivity is associated with higher risk of multiple mental health conditions and could be a potential maker in targeted prevention programs. While the reliable predictor in our study was a single measure of impulsivity from Substance Use Risk Profile Scale (SURPS) [46], it is worth noting that other studies have shown that various facets of impulsivity exhibit differential associations with depressive symptoms [47]. Further studies are needed to clarify whether specific facets of impulsivity are uniquely associated with particular mental health symptoms.

While being female and higher peer relationship problems were associated with future depressive symptoms, being male and lower peer relationship problems elevated risks of future harmful drinking. Although peer relationships consistently correlate with alcohol use in young people, evidence from longitudinal studies has been scarce and inconsistent [48,49]. Our result may reflect the role of alcohol consumption as a common means of harnessing and developing social connections. During social drinking occasions, factors related to one's image and reputation among peers are the main drivers of excessive drinking in young people [50], and other factors include coercion and fear of exclusion. In addition, close peer relationships can enhance feelings of safety toward drinking [51]. Our finding suggests that prevention and early intervention efforts can be enhanced by raising awareness on the social factors contributing to harmful drinking [52], in addition to its adverse impact on individual's health.

## Strengths and limitations

Our study has clear strengths, notably the combination of a clinical sample and a longitudinal, population-based cohort similarly assessed on a wide range of psychosocial domains. However, some limitations should be acknowledged. First, our ED sample involved women only. Also, our study involved predominantly Caucasian participants, therefore it remains to be tested how our findings generalize to other ethnic groups. Second, our study did not include some well-known risk factors of EDs such as perfectionism and cognitive inflexibility. Third, while our focus was on the top 10 most reliable features, it should be noted that features beyond the top 10 also made contributions, albeit to a less extent. Lastly, while the Elastic Net model offered high interpretability regarding how variables contribute to the outcome, the accuracies for the longitudinal prediction were not adequate for real-world clinical settings. Larger and enriched samples, and more powerful prediction techniques will be required in future studies to achieve better predictability.

## Conclusion

Our study demonstrates the capability of machine learning methods to accurately predict mental health diagnoses by leveraging multi-domain psychosocial data. The transdiagnostic nature of the classification models revealed shared features across a spectrum of disorders, encompassing AN, BN, MDD, and AUD, with notable contributions from neuroticism, hopelessness, and ADHD symptoms. Furthermore, the predictive models for future mental health symptoms successfully identified early predictors, emphasizing the roles of pubertal development, impulsivity, and peer relations in shaping the development of symptoms related to EDs, depression, and harmful drinking. These findings shed light on crucial aspects influencing mental health outcomes, providing a foundation for targeted prevention and interventions. As we advance our understanding, our work suggests the need for future studies with larger and enriched sample sizes to strengthen the predictive capabilities of machine learning in mental health, fostering a more nuanced and effective approach to diagnosis, intervention, and prevention strategies.

## Methods

#### Participants

Participants were assessed as part of the ESTRA, STRATIFY, and IMAGEN studies. These were sibling studies that were designed with matched assessments and protocols to enable comparability.

#### Case-control studies

Our clinical sample included participants with AN and BN, recruited as part of the ESTRA study. All the participants were female, aged 18-25 years, and recruited at the London study site. Healthy controls (HC) for the ED patients were selected from the IMAGEN study (see below) at the third follow-up (~23 years old), as being female, recruited in London, and screened negative for all psychiatric diagnoses based on the Mini International Neuropsychiatric Interview [53]. Participants with MDD and AUD, and the corresponding HCs were aged 18-25 years and recruited as part of the STRATIFY study from three study sites: London, Southampton, UK and Berlin, Germany. Written consent was obtained from all the participants (see Supplementary Methods for more details).

#### Longitudinal cohort study

This population sample was derived from IMAGEN, a longitudinal neuroimaging and genetics study of adolescents recruited from eight study sites in Europe [54]. Written assent was obtained from all the participants and written consent from their parents/guardians. The data used in the longitudinal prediction analysis were acquired at ages 14, 16, and 19 years.

Eating disorder symptoms were assessed by self-report of concerns over one's shape, weight, and eating, and disordered eating behaviors (binge-eating, purging, and dieting) based on the Development and Wellbeing Assessment (DAWBA) [55]. 'Developers' were defined as individuals who did not report any ED symptom at age 14, but reported one or more symptoms at ages 16 or 19. They were compared to controls, who remained asymptomatic across the three ages. Developers of depression and harmful drinkingwere defined as scoring low on depressive symptoms and harmful drinking [56], respectively, at age 14, but high at ages 16/19. Controls for these groups scored low on depressive symptoms and harmful drinking, respectively, across the three ages (for more details, see Supplementary Methods). Data collected at age 14 were used to predict whether participants developed each mental health symptom at ages 16 or 19.

#### Measures

Demographic information, including sex assigned at birth, age, and ethnicity was acquired from self-report. Our analyses combined a wide range of data domains comprising cognition, environment, personality, psychopathology, substance use, and BMI (for full details, see Supplementary Methods). Full lists of variables and percentages of missing data are provided in Supplementary Tables 2-4.

#### Data Analysis

A logistic regression model with L1 and L2 regularization, namely Elastic Net was used, implemented in the glmnet (version 4.1-7) package [57] in R (version 4.2.1). Model performance was assessed by area under the receiver operating characteristic curve (AUC-ROC) and area under the precision and recall curve (AUC-PR). These performance metrics were derived from a nested cross-validation (CV) procedure. The whole dataset was randomly split into 10 subsets. The ratio between cases and controls was maintained the same across these subsets. One subset (10% of the whole dataset) was reserved for model testing, and the remaining data (90% of the whole dataset) was used for model training.

The data preparation procedure included imputation of missing values, partialling out the effect of confounding variables, standardization, and dealing with extreme values. First, missing data were imputed in the training and testing data separately, by using a Random Forest-based method implemented in the missForest package [58] in R (version 4.2.1). Second, the effects of confounding variables were partialled out from the training and testing data separately, following the procedure recommended by Snoek et al. (2019) [59]. For each feature in the training data, a linear regression model was fitted with the confounding variables as the only predictors. Residuals from this model were used for model training. This linear regression model was directly applied to the testing data (without model refitting) to obtain residuals of each feature. This approach ensured that no information from the testing data was utilized in the model training process. Third, each feature in the training data was standardized into z-scores. The mean and standard deviation of each feature in the training data were used to standardize the testing data. Last, to mitigate the impact of extreme values on model fitting, the z-scores smaller than -3 or larger than 3 were recoded as -3 and 3, respectively.

A five-fold inner CV was nested in the training data to select the optimal hyper-parameters (alpha and lambda) for the Elastic Net model, with the goal of maximizing AUC-ROC on the training data. By using the optimal hyper-parameters, an Elastic Net model was fitted on all the training data (90% of the whole dataset). The classification performance of the constructed model was assessed using the remaining subset (10% of the whole dataset). This process was repeated until each subset had been used as the testing data. If the model involved a single predictor of BMI, an ordinary logistic regression model was used instead. The same 10-fold CV procedure was employed as above, but the nested CV and hyper-parameter tuning procedures were omitted.

The above CV procedure was repeated 10 times to mitigate the effect of data splitting. The model's performance metrics were averaged across the 10 repetitions. The ROC curves were plotted with the ROCR package (https://CRAN.R-project.org/package=ROCR).

*Sample weighting in the prediction models*: In building the prediction models using the longitudinal IMAGEN data, the model training and testing procedures were the same as those used for the clinical sample, except that sample weights were provided for the model training to deal with group size imbalances between the developers and controls (Supplementary Table 1). The weight of a sample was inversely proportional to the group size, thus assigning higher weights to the developers than the controls.

*Bootstrapping confidence intervals*: Confidence intervals of the performance metrics (AUC-ROC and AUC-PR) were obtained by using bootstrapping. For each repeat of the CV, the model's output was resampled with repetition. Based on the resampled values the performance metrics were obtained. This procedure was repeated 2000 times for each repeat of the CV, forming a bootstrap distribution. Lower and upper bounds of the CI were derived from 2.5% and 97.5% percentile of the bootstrapping distribution and averaged across the 10 repetitions.

*Permutation test*: P values for the model's performance were obtained from permutation tests. We randomly shuffled the group membership of samples before submitting the data to the same CV procedure described above, and derived performance metrics. This procedure was repeated 5000 times to derive null distributions of AUC-ROC and AUC-PR. To calculate the P-value, we counted how many values in the null distribution exceeded the actual performance and divided this count by the number of permutations.

*Classification of ED patients:* Firstly, we included all the variables (n=47) in building the classification model and considered age as a confounding variable. Given BMI is a diagnostic criterion for AN, a second model was run after excluding BMI. We further built models that involved each data domain alone to test if they could distinguish ED groups. A total of 18 models were built (Figure 1). A variable was identified as a reliable contributor to the Elastic Net model if it had a non-zero coefficient in at least 90% of all the CV folds [60]. The coefficient of the model for each feature was averaged across all the CV folds to obtain the median value, which represents the feature's importance.

*Classification of MDD and AUD patients*: We excluded 14 variables with excessive missing data, such as measures of cognitive performance and traumatic experiences (as indicated in Supplementary Table 3). Furthermore, we excluded depressive and emotional symptoms from MDD vs. HC analysis, and excluded the harmful drinking scale from AUD vs. HC analysis. Considering a sex bias in the HC group (59% females, Supplementary Table 1), sex was considered as a confounding variable, in addition to age and study site.

*Transdiagnostic models*: We tested whether the model derived from the AN vs. HC and BN vs. HC analyses could also distinguish MDD and AUD from HC, and vice versa. As we are aware, BMI is a diagnostic criterion of AN but is unrelated to MDD and AUD. Therefore, BMI was excluded from the transdiagnostic analysis. In addition, variables with excessive missing data in the AUD and MDD samples, such as measures of cognitive performance, traumatic experiences (as indicated in Supplementary Table 3), were also excluded. To derive a single model for AN vs. HC classification, we used the median values of the hyper-parameters (alpha and lambda) across all the CV folds to train a model using the entire AN and HC data. We tested whether this model could distinguish MDD and AUD from HC, respectively. Similarly, we trained a model for BN vs. HC classification and tested it on the MDD and AUD samples. Conversely, we tested whether the models developed for MDD vs. HC and AUD vs. HC classifications could classify ED patients from healthy controls. The same data preparation procedure was adopted from the classification analyses, including data imputation, adjustment for confounding variables, standardization, and handling extreme values.

*Predicting the development of future mental health symptoms*: The top 10 reliable variables identified from the classification analyses in the clinical EDs, MDD, and AUD samples were pooled together and used for the prediction analysis in the longitudinal population sample. Data collected at age 14 were used to predict the development of ED symptoms, depressive symptoms, and harmful drinking at ages 16/19 years. In addition, we built a second model by adding known risk factors of EDs, including sex, BMI, and pubertal development scale to investigate whether they could improve prediction accuracy.

## Declarations

All procedures involving human subjects/patients were approved for the ESTRA study by the North West-Greater Manchester South Research Ethics Committee in the UK. The STRATIFY study was approved by research ethics committees in the UK and Germany. The IMAGEN study was approved by the local research ethics committee at each study site (London, Nottingham, UK; Dublin, Ireland; Paris, France; Berlin, Hamburg, Mannheim, and Dresden, Germany). Written informed consent was obtained from all participants aged 18 years and above. For all participants under 18 years, written assent was obtained from them and written consent from their parents/guardians.

Acknowledgements

This work received support from the following sources: the Medical Research Council and Medical Research Foundation (grants MR/R00465X/1 and MRF-058-0004-RG-DESRI: ‘ESTRA: Neurobiological underpinning of eating disorders: integrative biopsychosocial longitudinal analyses in adolescents’; MR/S020306/1 and MRF-058-0009-RG-DESR-C0759: ‘Establishing causal relationships between biopsychosocial predictors and correlates of eating disorders and their mediation by neural pathways’), the Medical Research Council (grant MR/W002418/1: ‘Eating Disorders: Delineating illness and recovery trajectories to inform personalized prevention and early intervention in young people (EDIFY)’); Medical Research Foundation fellowship (MRF-058-0014-F-ZHAN-C0866) awarded to Zuo Zhang; The European Union-funded FP6 Integrated Project IMAGEN (Reinforcement-related behaviour in normal brain function and psychopathology) (LSHM-CT- 2007-037286), the European Union and Innovate UK funded project ‘environMENTAL’ (grants 101057429 and 10038599) the Horizon 2020 funded ERC Advanced Grant ‘STRATIFY’ (Brain network based stratification of reinforcement-related disorders) (695313), Human Brain Project (HBP SGA 2, 785907, and HBP SGA 3, 945539), the Medical Research Council Grant 'c-VEDA’ (Consortium on Vulnerability to Externalizing Disorders and Addictions) (MR/N000390/1), the National

Institute of Health (NIH) (R01DA049238, A decentralized macro and micro gene-by-environment interaction analysis of substance use behavior and its brain biomarkers), the National Institute for Health and Care Research (NIHR) Maudsley Biomedical Research Centre (BRC) at South London and Maudsley NHS Foundation Trust and King's College London, the Bundesministerium für Bildung und Forschung (BMBF grants 01GS08152; 01EV0711; Forschungsnetz AERIAL 01EE1406A, 01EE1406B; Forschungsnetz IMAC-Mind 01GL1745B), the Deutsche Forschungsgemeinschaft (DFG grants SM 80/7-2, SFB 940, TRR 265, NE 1383/14-1), the Deutsche Forschungsgemeinschaft (DFG, German Research Foundation) under Germany's Excellence Strategy – EXC-2049 – 390688087, the National Institutes of Health (NIH) funded ENIGMA (grants 5U54EB020403-05 and 1R56AG058854-01). Further support was provided by grants from: - the ANR (ANR-12-SAMA-0004, AAPG2019 - GeBra), the Eranet Neuron (AF12-NEUR0008-01 - WM2NA; and ANR-18-NEUR00002-01 - ADORe), the Fondation de France (00081242), the Fondation pour la Recherche Médicale (DPA20140629802), the Mission Interministérielle de Lutte-contre-les-Drogues-et-les-Conduites-Addictives (MILDECA), the Assistance-Publique-Hôpitaux-de-Paris and INSERM (interface grant), Paris Sud University IDEX 2012, the Fondation de l'Avenir (grant AP-RM-17-013), the Fédération pour la Recherche sur le Cerveau; the National Institutes of Health, Science Foundation Ireland (16/ERCD/3797), U.S.A. (Axon, Testosterone and Mental Health during Adolescence; RO1 MH085772-01A1), and by NIH Consortium grant U54 EB020403, supported by a cross-NIH alliance that funds Big Data to Knowledge Centers of Excellence.

The recruitment materials of the ESTRA study were reviewed by a team with experience of mental health problems and their carers who have been specially trained to advise on research proposals and documentation through the Young Person's Mental Health Advisory Group: a free, confidential service in England provided by the National Institute for Health and Care Research Maudsley Biomedical Research Centre via King's College London.

Conflict of interest disclosures

Dr Banaschewski served in an advisory or consultancy role for Lundbeck, Medice, Neurim Pharmaceuticals, Oberberg GmbH, Shire. He received conference support or speaker's fee by Lilly, Medice, Novartis and Shire. He has been involved in clinical trials conducted by Shire & Viforpharma. He received royalties from Hogrefe, Kohlhammer, CIP Medien, Oxford University Press. Dr Barker has received honoraria from General Electric Healthcare for teaching on scanner programming courses. Dr Poustka served in an advisory or consultancy role for Roche and Viforpharm and received speaker's fee by Shire. She received royalties from Hogrefe, Kohlhammer and Schattauer. M. John Broulidakis receives a salary from medical device manufacturer Emteq Labs for which he works as a research scientist. Emteq Labs had no role, financial or otherwise, in the STRATIFY or IMAGEN projects or this paper in particular. Views expressed in this paper do not necessarily reflect those of Emteq Labs. The present work is unrelated to the above grants and relationships. The other authors report no biomedical financial interests or potential conflicts of interest.

Data availability

De-identified data of the IMAGEN, STRATIFY, and ESTRA studies are available to researchers after approval of a proposal by the Executive Committee of these studies. The data proposal forms can be downloaded from https://imagen-project.org/the-imagen-dataset/ (IMAGEN) and https://stratify-project.org/the-stratify-dataset/ (STRATIFY and ESTRA).

## Figures

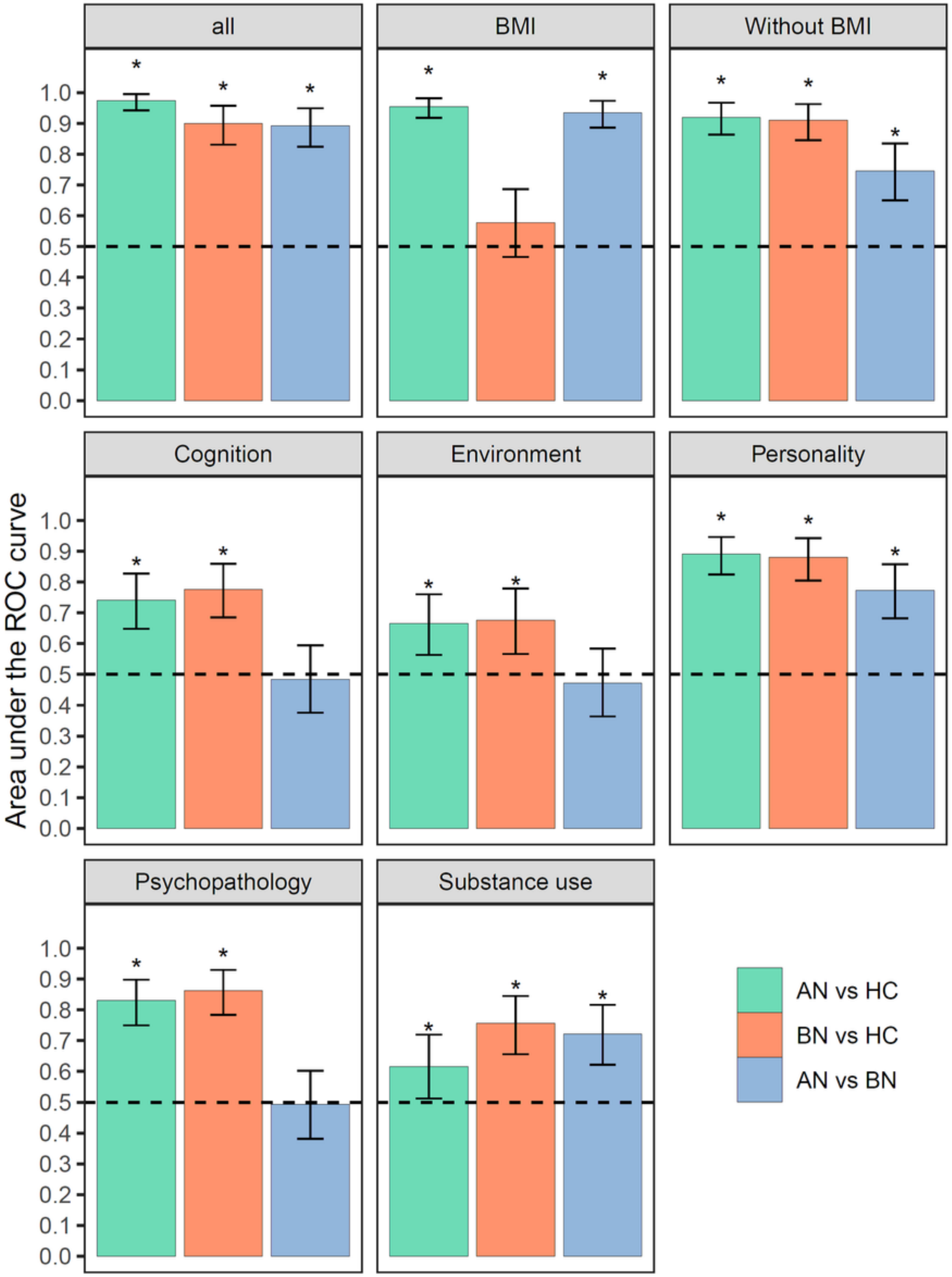

**Figure 1**

Classification performance on the AN, BN, and HC samples. Asterisks (*) indicate the performance is significantly above chance after correction with false discovery rate (FDR<0.05) for the 24 tests. Error bars indicate 95% bootstrap confidence intervals. Dashed lines indicate chance level performance (0.5). ROC curve, receiver operating characteristic curve. AN, anorexia nervosa. BN, bulimia nervosa. HC, healthy controls.

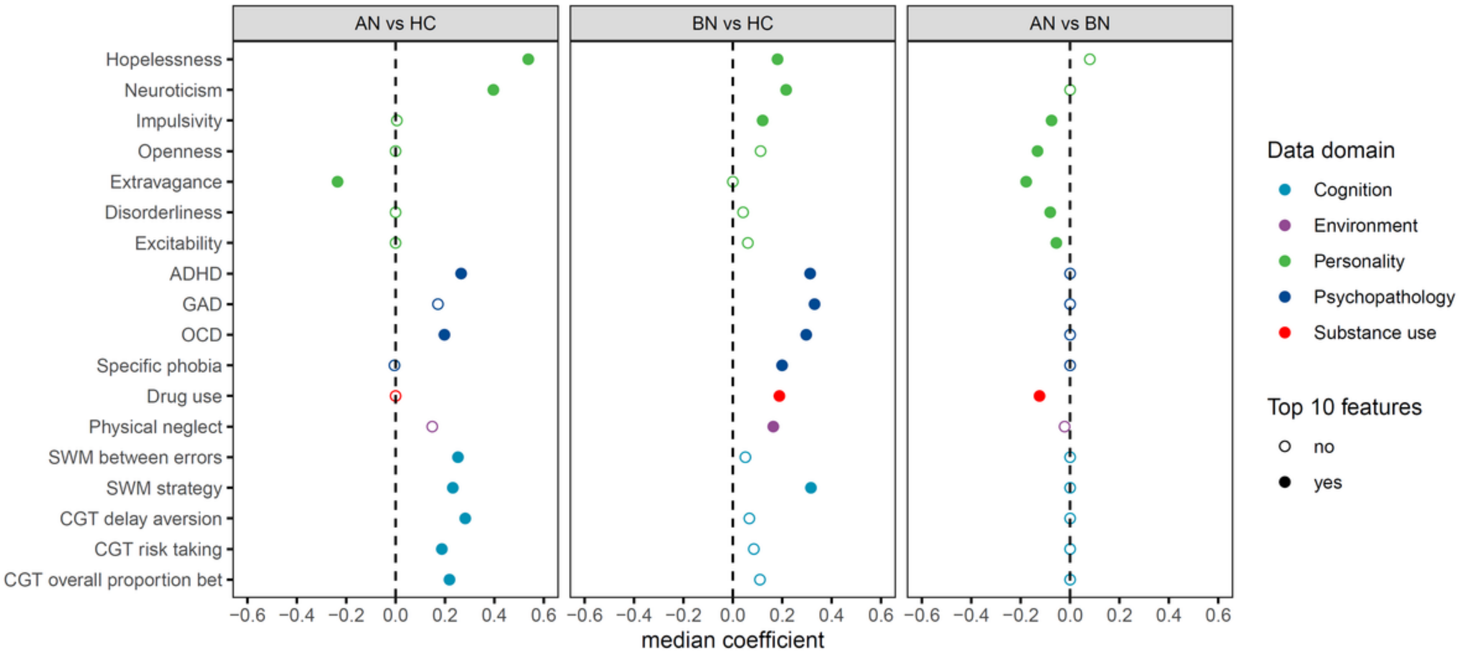

### Figure 2

Top 10 reliable features identified from the classification model that involved all data domains without BMI. Features are listed if they were among the top 10 reliable features for at least one analysis. Top 10 reliable features are indicated by solid circles. All the features were standardized as z-scores. Feature importance was measured by calculating the median value of the model coefficients across all the folds of cross-validation. AN, anorexia nervosa. BN, bulimia nervosa. HC, healthy controls. ADHD, Attention-deficit/hyperactivity disorder. GAD, Generalized anxiety disorder. OCD, Obsessive compulsive disorder. CGT, Cambridge gambling task. SWM, spatial working memory.

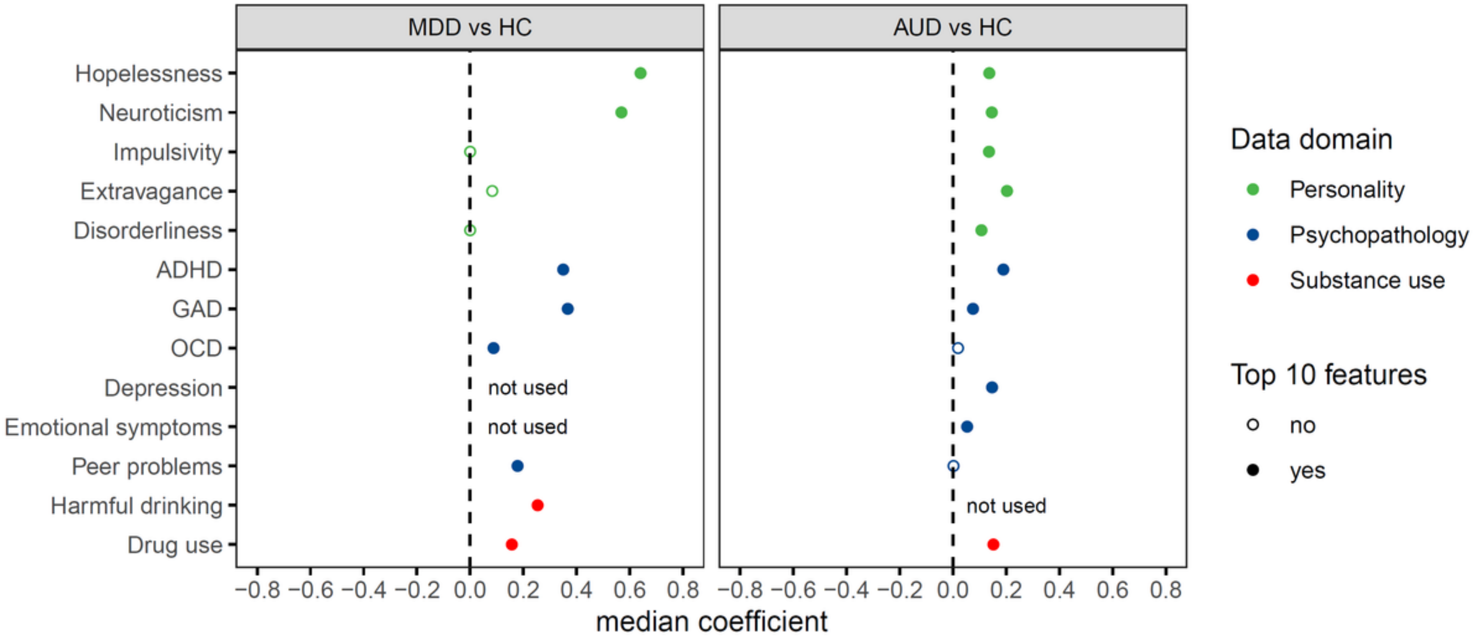

### Figure 3

Top 10 reliable features identified from the classification between MDD, AUD, and HC groups. Features are listed if they were among the top 10 reliable features for at least one analysis. Top 10 reliable features are indicated by solid circles. All the features were standardized as z-scores. AUD, alcohol use disorder. MDD, major depressive disorder. HC, healthy controls. ADHD, Attention-deficit/hyperactivity disorder. GAD, Generalized anxiety disorder. OCD, Obsessive compulsive disorder.

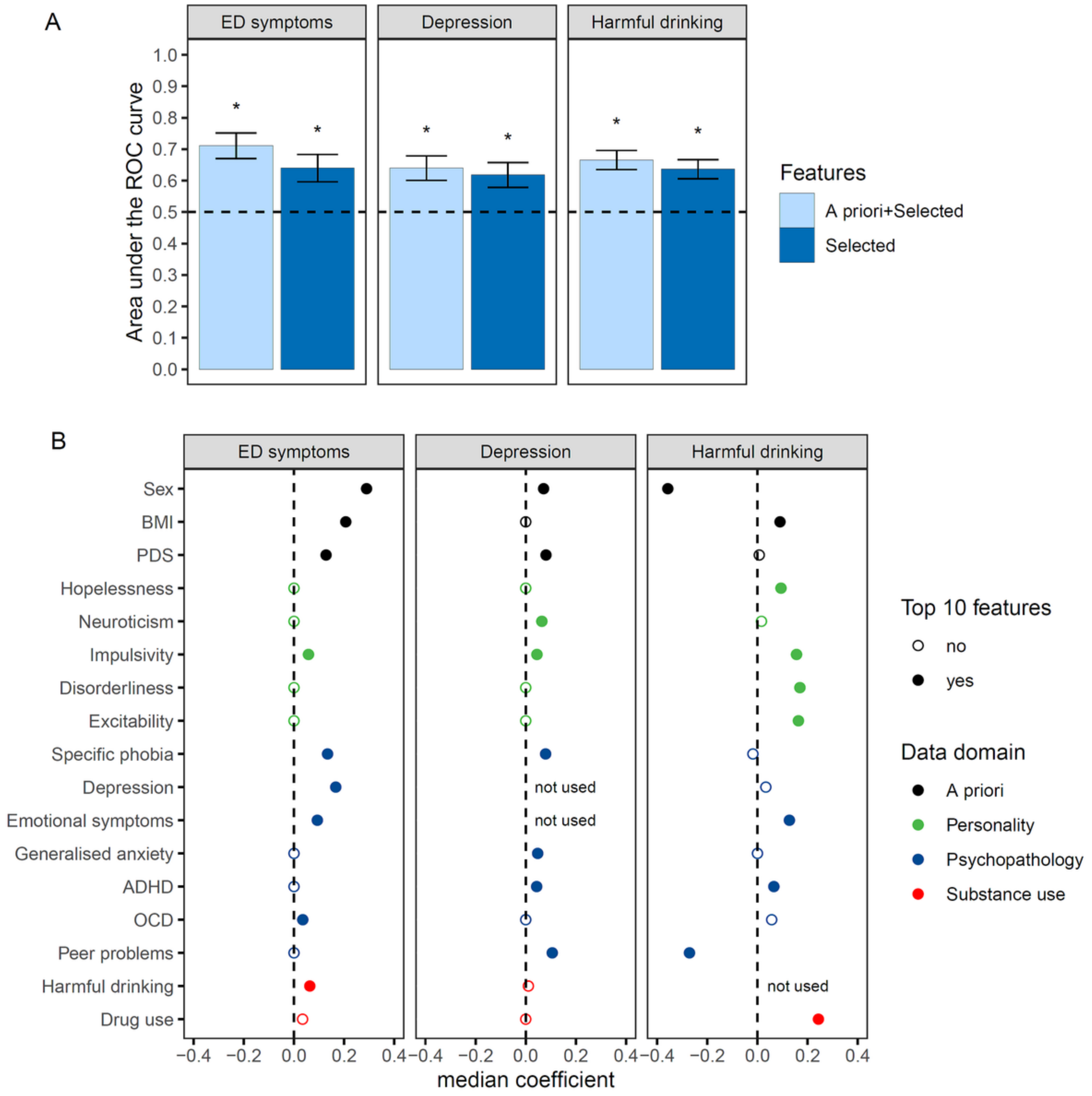

### Figure 4

Results of predicting the development of mental health symptoms (A) and top 10 reliable predictors (B). Features are listed if they were among the top 10 reliable features for at least one analysis. Top 10 reliable features are indicated by solid circles. All the features except sex were standardized as z-scores. ROC curve, receiver operating characteristic curve. PDS, pubertal development scale. ADHD, attention-deficit/hyperactivity disorder. OCD, Obsessive compulsive disorder.

## Supplementary Files

This is a list of supplementary files associated with this preprint. Click to download.

- MLmodelsupplementry20231219.docx
